# Effects of Tissue Preservation on Carbon and Nitrogen Stable Isotope Signatures in Syngnathid Fishes and Prey

**DOI:** 10.3390/ani10122301

**Published:** 2020-12-04

**Authors:** Miquel Planas, Alex Paltrinieri, Mario Davi Dias Carneiro, Jorge Hernández-Urcera

**Affiliations:** 1 Institute of Marine Research (CSIC), 36208 Vigo, Spain; alex.paltrinieri.acq@gmail.com (A.P.); marioiddc@gmail.com (M.D.D.C.); jurcera@iim.csic.es (J.H.-U.); 2 Dipartimento di Scienze della Vita e dell’Ambiente, Università Politecnica delle Marche, 60131 Ancona, Italy; 3 Laboratório de Piscicultura Estuarina e Marinha, Instituto de Oceanografia, Universidade Federal do Rio Grande—FURG, Rio Grande, RS 96203-900, Brazil

**Keywords:** stable isotopes, preservation, syngnathids, seahorses, pipefishes, conversion models

## Abstract

**Simple Summary:**

Stable isotope analysis (SIA) was used to assess the influence of various preservation methods (freezing, ethanol and formaldehyde) on syngnathid (seahorses and pipefishes) fins, seahorse newborns (seahorses), and prey (copepods and *Artemia*). The first available conversion models for syngnathids are provided, enabling their application to isotopic studies in the field and in the laboratory.

**Abstract:**

Isotopic stable analysis (SIA) is a powerful tool in the assessment of different types of ecological and physiological studies. For that, different preservation methods for sampled materials are commonly used prior to isotopic analysis. The effects of various preservation methods (freezing, ethanol and formaldehyde) were analyzed for C:N, and δ^13^C and δ^15^N signals on a variety of tissues including dorsal fins (three seahorse and two pipefish species), seahorse newborns (three seahorses species), and prey (copepods and different stages of *Artemia*) commonly used to feed the fishes under rearing conditions. The aims of the study were: (i) to evaluate isotopic effects of chemical preservation methods across different types of organisms and tissues, using frozen samples as controls, and (ii) to construct the first conversion models available in syngnathid fishes. The chemical preservation in ethanol and, to a lesser extent, in formaldehyde significantly affected δ^13^C values, whereas the effects on δ^15^N signatures were negligible. Due to their low lipid content, the isotopic signals in fish fins was almost unaffected, supporting the suitability of dorsal fins as the most convenient material in isotopic studies on vulnerable species such as syngnathids. The regression equations provided resulted convenient for the successful conversion of δ^13^C between preservation treatments. Our results indicate that the normalization of δ^15^N signatures in preserved samples is unnecessary. The conversion models should be applicable in isotopic field studies, laboratory experiments, and specimens of historical collections.

## 1. Introduction

Stable isotope compositions of carbon and nitrogen (typically expressed as δ^13^C and δ^15^N, respectively), are used in a variety of studies, including tracing trophic chains [1,2], estimation of trophic enrichment factors [1,2,3], diet reconstruction [4,5], tissue turnover rates [6,7], discrimination between hatchery-reared and wild spawned individuals, or migrations [8,9].

The isotopic composition of animal tissues reflects the dietary isotopic composition, especially for C and N, within a difference of a few units (discrimination factor) [10,11]. Isotopic signatures are species- and tissue-specific [12,13] and might vary depending on several factors such as dietary isotopic values or developmental stage [14,15,16]. However, some important issues on the application of stable isotope analysis (SIA) need to be properly addressed, particularly on type of tissue, sample preservation method, and lipid correction.

Whenever possible, SIA is performed on muscle tissue due to its relative isotopic stability [12]. Where lethal sampling is not desirable, other fish tissues such as fins and scales are non-lethal alternatives to muscle [15,17,18,19,20], especially in threatened and endangered species [21,22,23,24]. The isotopic signals from those surrogate tissues can be converted to muscle values by means of mathematical corrections obtained from inter-tissue comparisons.

The methods commonly used for tissue preservation include drying, freezing, ethanol or formaldehyde, depending on the objectives, limitations of the study and sampling conditions [25,26,27,28,29]. However, the results of inter-methodological comparisons might be unpredictable and vary among taxa, suggesting the need to analyze zoological groups separately [26,28].

Lipids are depleted (more negative values) in δ^13^C when compared to other biochemical compounds (e.g., proteins, carbohydrates), affecting bulk tissue signatures [10,11,30,31,32]. Lipid extraction may also result in changes in δ^15^N values of the lipid-free sample [33]. Alternatively, mathematical normalization techniques should be applied for lipid normalization when C:N ratios are high [34,35]. Ideally, corrections should be applied using model estimates on the same or similar type of organisms.

In the present study, we assessed the effects of freezing and solvent preservatives (ethanol and formaldehyde) on C:N ratios and δ^13^C and δ^15^N signatures both in syngnathid fishes, including adults (fin clipping) and newborns (bulk specimens), and in prey (copepods and different stages of *Artemia*) commonly used to raise those fishes. Syngnathids are a family of fishes including endangered and vulnerable species [36,37]. For that reason, the use of lethal sampling should be avoided whenever possible, and fin tissue is an excellent and suitable material for isotopic analyses [22]. Since tissue conversion models specific for syngnathid fishes were lacking, the main aim of this study was to provide for the first time mathematical corrections for δ^13^C and δ^15^N in syngnathid species considering different types of specimens and preservation methods. The models provided would be helpful in field, experimental and natural history collections.

## 2. Materials and Methods

### 2.1. Live Prey

We analyzed different sources of prey commonly used in the rearing of syngnathids: calanoid copepods (*Acartia tonsa*) and *Artemia* (nauplii, metanauplii enriched for 24 and 72 h, enriched adults and unenriched adults). Microalgae (*Phaeodactylum tricornutum*, *Rhodomonas lens* and *Isochrysis galbana*) were cultivated at 22 ± 1 °C on F2P media to feed copepods *Acartia tonsa* and *Artemia* until the adult stage. The copepods were fed on the microalgae *R. lens* (26–27 °C and 38 salinity). Only copepods retained on a 180 µm mesh (copepodites and adults) were analyzed.

*Artemia* nauplii and metanauplii were produced to feed seahorse juveniles, whereas adult *Artemia* were delivered to adult seahorses. *Artemia* cysts (EG MC450 and AF; Ocean Nutrition, San Diego, CA, USA) were hatched at 28 °C and newly hatched nauplii were collected to produce enriched metanauplii (from AF cysts) and adults (from EG cysts). For metanauplii, the nauplii were enriched (2–3 days at 100 *Artemia* mL^−1^) twice daily on a mixture including live microalgae *P. tricornutum*, Red Pepper (Bernaqua, Belgium) and dried *Spirulina*.

The production of adult *Artemia* was carried out at 26–28 °C. The adults were long-time enriched (3–6 days) or unenriched. The enrichment was carried out as previously reported [38].

All samples were rinsed with distilled water, preserved according to the established procedures (see below), dried for 48 h (60 °C) and manually homogenized using a mortar and pestle.

### 2.2. Fishes

The following five species of Syngnathidae were analyzed: pipefishes *Syngnathus acus* Linnaeus, 1758 and *Syngnathus typhle* Linnaeus, 1758, and seahorses *Hippocampus guttulatus* Cuvier, 1829, *Hippocampus hippocampus* Linnaeus, 1758 and *Hippocampus reidi* Ginsburg, 1933. The pipefishes were captured in Arcade Cove (Ría de Vigo, NW Spain) in March–April 2016, transferred to the laboratory and fin clipped for further sampling. The seahorses were reared in captivity and sampled for dorsal fin tissue (fin clipping) and bulk newborn. The adults were fed on mixtures of adult *Artemia* and mysidaceans (commercial frozen *Neomysis* sp., and live wild caught *Siriella armata* and *Leptomysis* sp. Further details on the maintenance and rearing conditions for the three seahorse species were provided in [39,40].

A variable number (n > 5 per sample) of bulk juveniles were sampled for C:N and stable isotope analysis (SIA), and pooled prior to conservation. Newborn seahorses were sampled after the male’s pouch release (prior to first feeding) and euthanized with Tricaine MS-222 (0.1 mg L^-1^, Sigma Aldrich, Darmstadt, Germany).

### 2.3. Experimental Preservation Procedures

Tissue samples were submitted to direct freezing at −80 °C (control) or preserved in 95% ethanol (EtOH) or 4% formaldehyde (CH_2_O) (Merck, Germany) for comparisons on C:N ratios, and δ^13^C and δ^15^N values. Samples included prey (see above) and dorsal fins in five species of adult syngnathids (*H. guttulatus*, *H. hippocampus*, *H. reidi*, *S. acus* and *S. typhle*). Additionally, the bulk newborn of three seahorse species (*H. abdominalis, H. guttulatus* and *H. reidi*) were also collected and preserved and analyzed as for dorsal fins.

**Figure 1:**
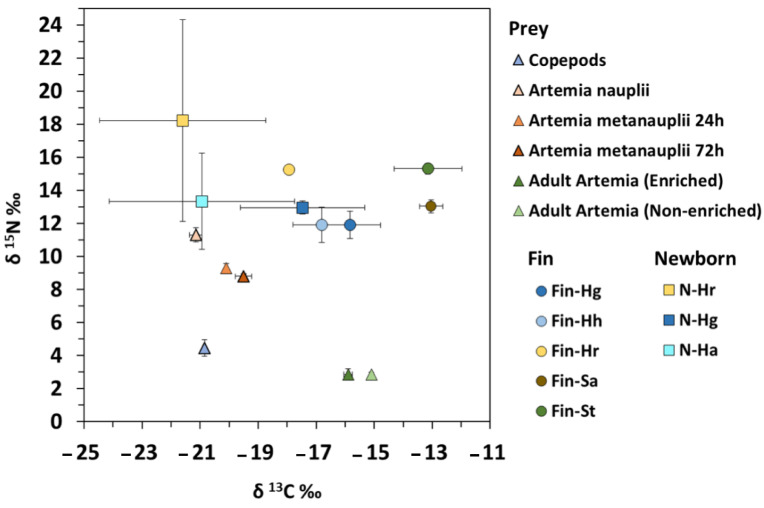
Stable isotope biplot of means (±SD) for δ^13^C and δ^15^N in prey, dorsal fins in adult syngnathids and bulk newborn seahorses. Values correspond to frozen samples. Ha—*Hippocampus abdominalis*; Hg—*H. guttulatus*; Hh—*H. hippocampus*; Hr—*H. reidi*; Sa—*Syngnathus acus*; St—*S. typhle*.

Seahorse breeders were fed on different types of prey (mysidaceans and adult *Artemia*), which affected isotopic signals in both fins and newborn. Consequently, samples tissues comprised a wide range in isotopic values (especially for δ^13^C) and C:N ratios, as shown in Figure 1.

All collected samples were filtered, rinsed with distilled water and stored using the different preservation procedures for 3–4 months prior to analysis.

### 2.4. Isotope Ratio Mass Spectrometry

Samples for stable isotope analyses (SIA) were homogenized and aliquots were transferred to preweighted tin capsules (ø 3.3 × 5 mm, 0.03 mL) (Lüdiswiss, Flawil, Switzerland). The analyses were made on sub-samples of 0.5–1 mg dry weight biomass. δ^13^C and δ^15^N values and elemental composition (total C and N percentage) were analyzed at Servizos de Apoio á Investigación (SAI) of the University of A Coruña (Spain) [22]. The samples were measured by continuous flow isotope ratio mass spectrometry using a FlashEA1112 elemental analyzer (ThermoFinnigan, San José, CA, USA) coupled to a Delta Plus mass spectrometer (FinniganMat, Bremen, Germany) through a Conflo II interface. Carbon and nitrogen stable isotope abundance was expressed as permil (‰) relative to VPDB (Vienna Pee Dee Belemnite) and Atmospheric Air, according to the following equation:

δX = (R_sample_/R_reference_) − 1,

where X is ^13^C or ^15^N and R is the corresponding ratio of ^13^C/^12^C or ^15^N/^14^N. As part of an analytical batch run, a set of international reference materials for δ^15^N values (IAEA-N-1, IAEA-N-2, USGS25) and δ^13^C values (NBS 22, IAEA-CH-6, USGS24) were analyzed. The range of C:N ratios in sampled tissues (2.9–6.3) were within the range (0.4–6.9) of reference materials used. The precision (standard deviation) for the analysis of δ^13^C and δ^15^N of the laboratory standard (acetanilide) was ±0.15‰ (1-sigma, n = 10). Standards were run every 10 biological samples. The isotopic analysis procedure fulfils the requirements of the ISO 9001 standard. The laboratory is submitted to annual intercalibration exercises (e.g., Forensic isotope ratio mass spectrometry scheme—FIRMS, LGC Standards, UK).

### 2.5. Data Analysis

The analyses were performed with R v.3.6.1 [41] and Statistica 8.0 packages (StatSoft, USA). The significance level was set at *p* < 0.05. The datasets on C:N and isotopic values in prey and seahorses (fins and newborn) were submitted for a Shapiro-Wilk test to confirm the assumption that the data were normally distributed, followed by analysis of covariance (ANCOVA) with organisms as covariates [42]. Covariates included in the analyses were prey type (copepods and several stages of *Artemia*), syngnathid genera (*Syngnathus* and *Hippocampus*), or seahorse species (*H. guttulatus, H. abdominalis* and *H. reidi*), depending on the analysis performed. Adjusted group means were obtained after partialing out the effects of the covariate using the Effect package in R. When significant, differences of means were submitted to multiple mean comparisons [43]. Least squares linear regression with 95% confidence intervals corrected against control values was used to assess the efficacy of the chemical preservation treatments. Regression models obtained with or without interception were compared using AIC (Arkaike Information Criterion). The models with the smallest AIC values were retained.

### 2.6. Bioethical Approval

Animal capture, handling and sampling were conducted in compliance with all bioethics standards on animal experimentation of the Spanish Government (Real Decreto 1201/2005, 10th October) and the Regional Government Xunta de Galicia (REGA ES360570202001/15/FUN/BIOL.AN/MPO01 and ES360570202001/16/EDU-FOR07/MPO01).

## 3. Results

The range of C:N values in controls (frozen samples) was 3.7–6.1 (mean: 4.8 ± 0.8) in prey, 2.9–3.5 (3.1 ± 0.2) in fish fins and 3.5–3.9 (3.7 ± 0.1) in fish newborn. Isotopic values in prey ranged from −21.1 to −14.7‰ for δ^13^C and from 1.6 to 11.1‰ for δ^15^N, with mean values of −18.7 ± 2.5‰ and 6.2 ± 3.6‰, respectively (Figure 1).

### 3.1. Effect on Carbon and Nitrogen Ratios

**Figure 2:**
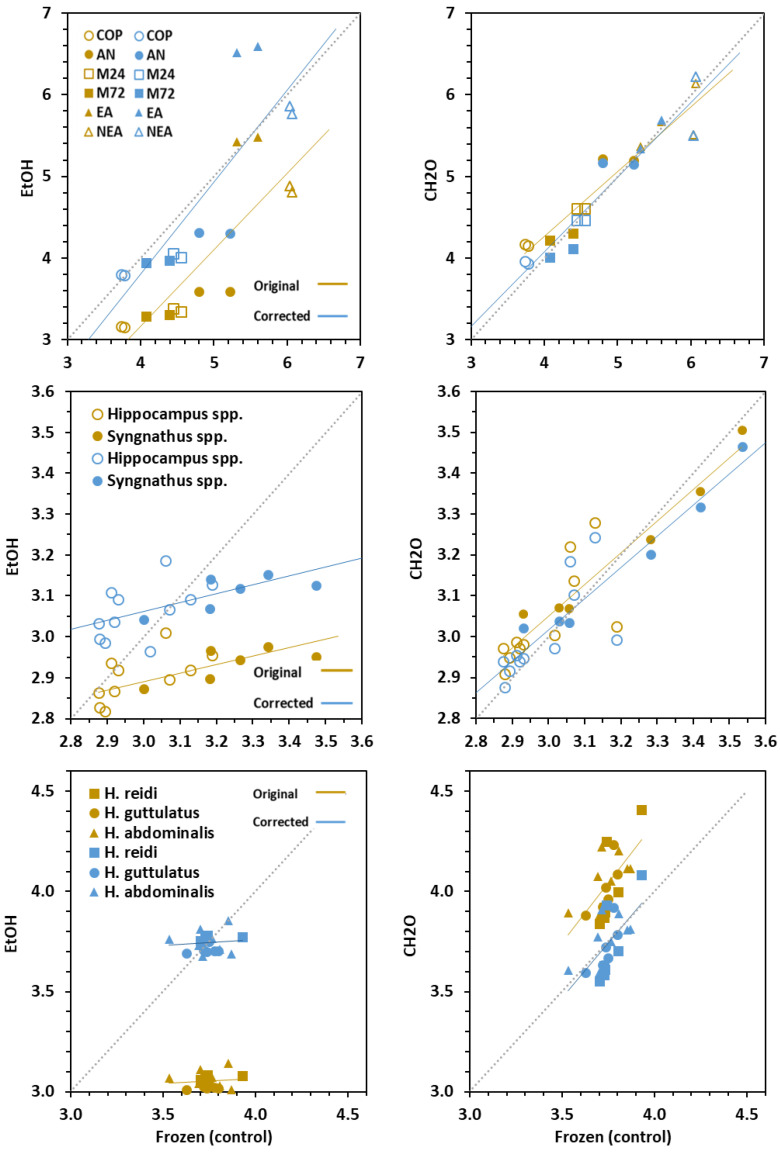
Original (brown symbols) and corrected (blue symbols) values for C:N in prey, fins and newborns. Regression lines are given for frozen, EtOH (ethanol) and CH_2_O (formaldehyde) relationships. A 1:1 dashed line (slope = 1, intercept = 0) is given for clarity. Prey: COP—copepods, AN—*Artemia* nauplii, M24 and M72—*Artemia* metanauplii, and; EA and NEA—enriched and non-enriched adult *Artemia*. Fin: genera *Syngnathus* and *Hippocampus*; Seahorse newborns: *H. guttulatus*, *H. abdominalis* and *H. reidi*.

Preservation treatments (freezing, EtOH and CH_2_O) of experimental samples (prey, fins and newborns) differed significantly (ANCOVA, *p* < 0.001) in their effect on C:N values (*p* < 0.081) (Table 1). When comparing to control groups (freezing), C:N values decreased significantly (*p* < 0.001) in EtOH-treated samples (Δ = −0.88 in prey; −0.15 in fins and −0.69 in newborn), whereas the effect of CH_2_O was minimal (Δ = 0.10 in prey and, 0.03 in fins) (*p* > 0.05), except in newborn seahorses (Δ = 0.29) (*p* < 0.001) (Table 1; Figure 2).

Considering dorsal fins, adjusted means for C:N values in *Hippocampus* (2.99) and *Syngnathus* (3.14) differed significantly (*p* = 0.001), even though the difference was small. In seahorse newborn, mean C:N values were similar (*p* = 0.506), ranging from 3.59 in *H. guttulatus* to 3.64 in *H. abdominalis*.

### 3.2. Effect on δ^13^C Signatures

**Figure 3:**
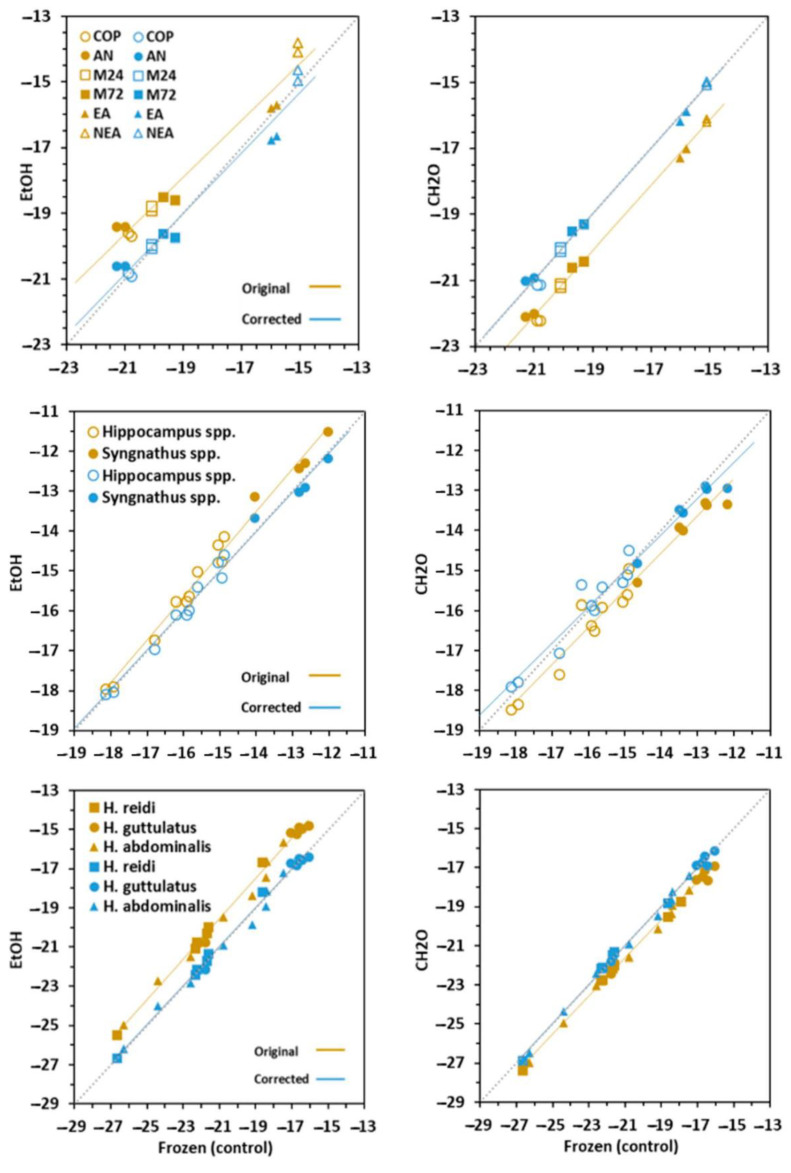
Original (brown symbols) and corrected (blue symbols) values for δ^13^C in prey, fins and newborns. Regression lines are given for frozen, EtOH (ethanol) and CH_2_O (formaldehyde) relationships. A 1:1 dashed line (slope = 1, intercept = 0) is given for clarity. Prey: COP—copepods, AN—*Artemia* nauplii, M24 and M72—*Artemia* metanauplii, and; EA and NEA—enriched and non-enriched adult *Artemia*. Fin: genera *Syngnathus* and *Hippocampus*; Seahorse newborns: *H. guttulatus*, *H. abdominalis* and *H. reidi*.

The signatures for δ^13^C in prey, fins and newborns was highly affected (*p* < 0.005) by chemical preservation (Table 1, Figure 3). The effects were directly related to C:N values in bulk frozen tissues. Accordingly, prey and fins were more (*p* < 0.001) and less (*p* = 0.031) affected, respectively. The isotopic signal increased (enriched) significantly (*p* < 0.001) in EtOH-treated samples (Δ = 1.1‰ in prey; 1.0‰ in newborn), except in clipped fins (Δ = 0.2‰) (*p* = 0.192). The treatment with CH_2_O led to decreased δ^13^C signals in prey (Δ = 1.1‰) (*p* < 0.001) but not in fish tissues (Δ = 0.0‰ in fins and 0.7‰ in newborns) (*p* = 0.365 and 0.391, respectively). Broadly, the effects of preservation methods in newborn seahorses were similar to those in fins.

Considering prey, adjusted means for δ^13^C were −20.9‰ in copepods and *Artemia* nauplii, −20‰ to −19.5‰ in *Artemia* metanauplii and −15.1‰ in non-enriched adult *Artemia*, and −16.3‰ in adult enriched *Artemia*. Isotopic signals for ^13^C in fish fins were −16.3‰ in *Hippocampus* and −13.3‰ in *Syngnathus*. Adjusted means in seahorse newborns were −17.2‰ in *H. guttulatus*, −19.5‰ in *H. abdominalis* and −21.0‰ in *H. reidi*.

### 3.3. Effect on δ^15^N Signatures

**Figure 4:**
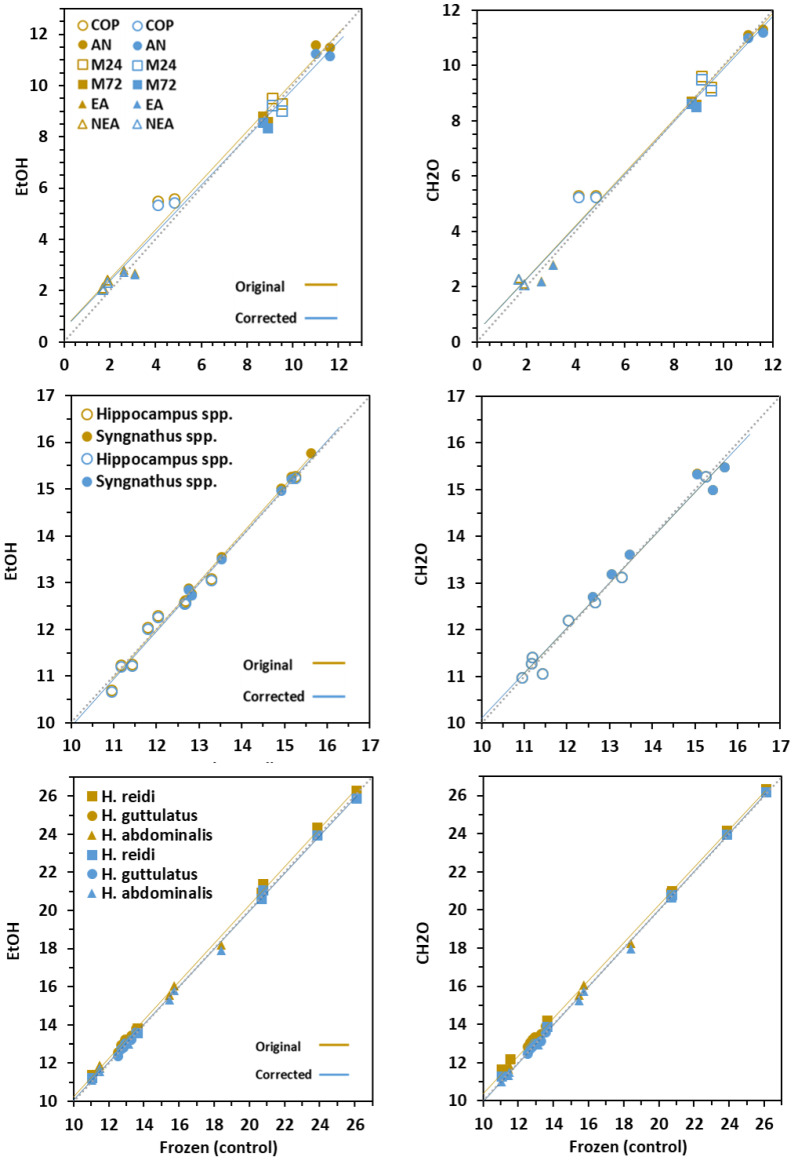
Original (brown symbols) and corrected (blue symbols) values for δ^15^N in prey, fins and newborns. Regression lines are given for frozen, EtOH (ethanol) and CH_2_O (formaldehyde) relationships. A 1:1 dashed line (slope = 1, intercept = 0) is given for clarity. Prey: COP—copepods, AN—*Artemia* nauplii, M24 and M72—*Artemia* metanauplii, and; EA and NEA—enriched and non-enriched adult *Artemia*. Fin: genera *Syngnathus* and *Hippocampus*; Seahorse newborns: *H. guttulatus*, *H. abdominalis* and *H. reidi*.

Chemical treatments performed similarly to frozen samples, and the effects on δ^15^N values were negligible, especially in fins (Δ < 0.2‰) and newborns (Δ < 0.3‰) (*p* < 0.001) (Table 1, Figure 4). The highest differences were achieved in prey (Δ = 0.1‰ for EtOH; Δ = 0.3‰ for CH_2_O).

Adjusted means for δ^15^N in prey ranged from 2.7‰ in copepods and 20‰ to −11.3‰ in *Artemia* nauplii. Dorsal fins in *Hippocampus* and *Syngnathus* fishes differed in isotopic adjusted means (12.4 and 14.2‰, respectively). Adjusted means for δ^15^N in newborns were 12.5‰ in *H. abdominalis,* 13.2‰ in *H. guttulatus* and 20.7‰ in *H. reidi*.

### 3.4. Conversion Models

The correction equations to account for changes of C:N, δ^15^N and δ^13^C values in tissues treated with chemical preservatives relative to freezing treatment are provided in Table 2 and visualized in Figure 2, Figure 3 and Figure 4. Corrected values in prey revealed worse adjustment and predictability compared to fish tissues. Except for C:N in CH_2_O-preserved newborns (Adj R^2^ > 0.384), the models were highly significant (Adj R^2^ > 0.9; range = 0.894–0.999). The main discrepancies between the original and corrected models occurred in C:N values, especially in EtOH–treated samples.

Simple arithmetic corrections could be applied to δ^15^N and δ^13^C values in fins and newborns for treatment correction. Overall, isotopic corrected lines for both chemical preservatives did not differ from the 1:1 line. Besides this, the slopes in most uncorrected and corrected linear models did not differ significantly, except for δ^13^C in prey and fins. On the contrary, regression intercepts were significantly different in many cases. However, the differences were highly reduced (<0.01) in δ^15^N models, and corrected and control values were statistically undistinguishable.

## 4. Discussion

The present study demonstrates for the first time the effects of freezing and two chemical preservation methods (ethanol—EtOH and formaldehyde—CH_2_O) on isotopic signals (δ^13^C and δ^15^N) and C:N ratios in syngnathid fishes, including adults (dorsal fin), and newborn juveniles, and in prey commonly used to feed marine fishes in rearing systems. Considering the preservation methods tested, EtOH and CH_2_O are polar and non-polar solvents frequently used as preservation chemicals. Formaldehyde hydrolyzes proteins and systematically affects δ^13^C signatures [44,45,46]. Even though EtOH does not remove lipids completely, the solvent is capable of extracting many fats, including phospholipids and free fatty acids. The global effects of each preservation method tested implied important differences across treatments depending on the type of tissue considered. CH_2_O-treated materials were more similar to control samples than those preserved in EtOH, especially for C:N values. Correcting C:N ratios and isotope signatures in chemically preserved tissues with derived correction equations revealed significant differences for C:N and δ^13^C values between treated and control (freezing) samples. The easiest, fastest and most economical procedure for SIA in adult syngnathids in laboratory experiments would be freezing of clipped-fin tissue, whereas EtOH (does not requires defatting) or CH_2_O-treatments would be recommended in the field to ensure better sample maintenance during sampling.

Ethanol led to a sharp but calculable decrease in C:N values. The magnitude of observed drop across tissues was directly correlated (Pearson’s R = 0.84) with C:N values (i.e., lipid content) in controls. Hence, lower C:N ratios in bulk tissues (fins) led to lower shifts in EtOH-treated samples (−0.2, −0.7 and −0.9 in fins, newborn and prey, respectively). Corrections on C:N ratios were reliable in prey but ineffective in fish tissues due to the low ratio variability derived from chemical treatments.

As compared to ethanol, formaldehyde-treated materials provided small shifts and low variability, affecting especially C:N values (all tissues) and δ^13^C signals. The depletion in ^13^C varied depending on the type of tissue, but prey and newborn were mostly affected (−1.1, −0.7 and −0.2‰ in prey, newborn and fin, respectively). Those changes would be concordant with tissue lipid content [25] and/or tissue formalin uptake [35,44].

Considering the average shifts achieved in δ^13^C signatures in fins (Δ < 0.2‰) and newborns (Δ < 1.0‰), we advise application of further corrections in chemical preserved tissues. On the contrary, δ^15^N signatures in solvent preserved materials were small enough (Δ < 0.2‰ in fins and <0.3‰ in newborns) to correct the values. Interestingly, average δ^15^N-shifts in both isotopes were much lower than the estimated trophic enrichment factor of 3.4‰ in aquatic animals [3] and 3.9–4.2‰ in adult syngnathids [23]. Hence, differences in δ^15^N between frozen and solvent preserved tissues will not affect data interpretation in food web studies for syngnathids.

The diverse tissues of an animal differ in turnover rates due to inter-tissue differences in isotopic fractionation [10,11,12,47,48,49]. Muscle tissue is the focal material in many isotopic studies due to its intermediate and less variable turnover rates [12,49]. The disadvantage of sampling fish muscle is that small specimens must be sacrificed [50]. However, there are alternative tissues that do not entail fish killing, providing isotopic signatures highly correlated to those in muscle [15,45,51]. Compared to many teleost, syngnathids are small fishes and their fins are difficult to sample, especially in small specimens. Consequently, the most convenient tissue for sampling is dorsal fin. However, differences might arise comparing dorsal fins with other fin tissues.

Fish fins are excellent tissues for isotopic assessment, especially in studies involving threatened or endangered species [17,22]. Fin clipping has been successfully used for SIA in syngnathids, both in field collected samples [23] and *ex situ* experimental studies [24]. Partial fin clipping results are advantageous when compared to other not detrimentally sampled tissues such as the dorsal fleshy filaments present in some seahorse species (e.g., *H. guttulatus* and *H. kuda*). Clipped filaments provide enough biomass for DNA analysis [39,52,53] but not for SIA. Fin and filament clipping does not impair fish behaviour [52,54] and both tissues are able to regenerate [53]. However, clipped filaments regenerate more slowly, and can recover histologically but not completely in size [52,53].

There is a high availability of isotopic studies on the effects of preservation methods and lipid normalization procedures in fish tissues [31,46,55,56]. Lipids are depleted in ^13^C when compared to proteins and carbohydrates [32]. Consequently, the main effect of EtOH preservation on *δ*^13^C of tissues is the loss of lipids and/or proteins, resulting in ^13^C enrichment [55]. In agreement with those findings, our EtOH-treated tissues were enriched in ^13^C, especially in materials with high C:N values (prey and newborn, in increasing order). However, other studies reported different results [25,44].

Even though lipids mainly affect the heavier ^13^C isotope, lipid extraction might also result in deviations in the δ^15^N of the lipid-free tissue [12,57,58]. Although there is a large variability, lipid extracts may be more depleted in ^15^N than bulk tissues. Commonly, δ^15^N signals in bulk tissues increase with trophic levels of organisms [33], as reflected in the present study when comparing prey with fish tissues (*δ*^15^N_prey_ < *δ*^15^N_fin_ > *δ*^15^N_newborn_). However, the preservation in EtOH did not affect *δ*^15^N signals, suggesting that lipids in all tissues analysed were highly ^15^N-depleted, and that storage in those solvents will not impair the reliability of the analyses.

Impaired δ^15^N values might also result due to artifacts in the extraction of lipids [5]. In the present study, we did not assess the effects of complete lipid extraction usually applied to samples with C:N ratios above 3.5 [34,56]. In this regard, it is likely that EtOH samples be isotopically equivalent to lipid-extracted bulk samples. A previous study carried out in seahorses comparing dorsal fin, muscle, and liver tissues reported similarities between δ^13^C and δ^15^N values in dorsal fin and muscle tissue, and significant effects of lipid extraction on δ^13^C values in muscle and liver [22]. The study concluded that lipid removal was not necessary in dorsal fin tissues due to a lipid content (2.6% dry weight) lower than in muscle tissue (7.1%). The low lipid content in fin tissues of syngnathids was confirmed by C:N values in the present study (2.88–3.19 in *Hippocampus* spp.; 2.93–3.53 in *Syngnathus* spp.). Accordingly, fin clipped samples could be submitted to SIA without the need of previous lipid extraction nor further mathematical lipid corrections for δ^15^N, whereas the regression models given in Table 2 should be applied for δ^13^C.

The main aim of the present study was to provide practical mathematical models for the conversion of isotopic signals in preserved live prey and syngnathids. The regression equations provided would be useful in field studies when samples cannot be properly stored, requiring preservation until further analysis. In addition, in spite of further assessment on the effect of long-term storage of sampled tissues on isotopic signals [25], the models might be applied to syngnathid-preserved specimens in natural history or museum collections. With regard to field studies in syngnathids, our study on adults was performed with samples collected both in the field and in cultivated fishes (seahorses vs. pipefishes) and both types responded similarly to solvents. An important feature in syngnathids is that fin tissues are really thin (with a low lipid content) compared to other large fishes, and it is likely that the results would be the same, whatever the scenario considered.

## 5. Conclusions

The results achieved revealed different effects of chemical preservation on isotopic signatures and C:N ratios both in syngnathid fishes (dorsal fin in adults and bulk newborns) and in prey commonly used to feed those fishes in the laboratory. Considering that dorsal fins are valid isotopic subrogates of muscle tissue in syngnathids, our results would be comparable to those in muscle tissue samples. However, the effects of feeding activity on potential differences between both tissue types deserve further consideration [59]. The impacts of ethanol were higher than those of formaldehyde, especially for δ^13^C signals and C:N ratios, but the effects of the former were consistent and predictable, and can be corrected. Conversely, the shifts in δ^15^N of chemically preserved tissues were small enough to be ignored. Hence, both solvents provided consistent and reliable results. The first conversion models for the mathematical correction of data across the tested preservatives were constructed specifically for syngnathids. Those taxa-specific models may be applied to field collected samples as well as to historical collections. Further work should be conducted to determine the isotopic effect of lipid extraction and duration of preservation, except in dorsal fins.

## Figures and Tables

**Table 1:** Summary of ANCOVA and pairwise comparisons for C:N, δ^15^N and δ^13^C values in organisms/tissues submitted to three preservation treatments (FR—frozen; ET—ethanol; FO—formaldehyde). Significant *p*-values are given in bold. Prey: copepods and *Artemia* (several developmental stages); Fin: genera *Syngnathus* and *Hippocampus*; Seahorse newborns: *H. guttulatus*, *H. abdominalis* and *H. reidi*.

		ANCOVA					Group Comparisons (*p*)		
	Effect	SS	d.f.	MS	F	Adj. *p*	FR–ET	FR–FO	ET–FO
Prey									
C:N	Treatment	6.90	2	3.45	41.43	**<0.001**	**<0.001**	0.403	**<0.001**
	Prey	18.83	5	3.77	45.19	**<0.001**			
	Residuals	2.33	28	0.08					
*δ*^13^C	Treatment	28.39	2	14.19	199.00	**<0.001**	**<0.001**	**<0.001**	**<0.001**
	Prey	186.41	5	37.28	522.80	**<0.001**			
	Residuals	2.00	28	0.07					
*δ*^15^N	Treatment	0.50	2	0.24	2.75	0.081	**0.027**	0.311	0.201
	Prey	435.30	5	87.05	990.40	**<0.001**			
	Residuals	2.50	28	0.09					
Fins									
C:N	Treatment	0.570	2	0.28	13.01	**<0.001**	**<0.001**	0.384	**<0.001**
	Genus	0.310	1	0.31	14.15	**<0.001**			
	Residuals	1.206	55	0.02					
*δ*^13^C	Treatment	9.26	2	4.63	3.70	**0.031**	0.192	0.365	**0.037**
	Genus	127.92	1	127.92	102.11	**<0.001**			
	Residuals	68.90	55	1.25					
*δ*^15^N	Treatment	0.38	2	0.19	0.10	**0.901**	0.712	0.718	0.998
	Genus	42.74	1	42.74	23.67	**<0.001**			
	Residuals	92.09	55	1.81					
Newborns									
C:N	Treatment	7.37	2	3.68	332.51	**<0.001**	**<0.001**	**<0.001**	**<0.001**
	Species	0.01	2	0.01	0.69	0.506			
	Residuals	0.443	40	0.010					
*δ*^13^C	Treatment	36.75	2	18.38	4.74	**0.014**	**0.040**	0.391	**0.005**
	Species	118.48	2	59.24	15.29	**<0.001**			
	Residuals	154.99	40	3.87					
*δ*^15^N	Treatment	1.00	2	0.49	0.05	**0.955**	0.801	0.787	0.986
	Species	613.90	2	306.96	28.38	**<0.001**			
	Residuals	427.30	40	10.68					

**Table 2:** Summary of least-square linear regression of C:N, δ^15^N and δ^13^C data and preservation methods (FR—frozen; ET—ethanol; FO—formaldehyde) across organisms (dorsal fin in adult syngnathids and bulk newborn seahorses). S, I—Original and corrected regression models: Significant differences in slopes (S) and intercepts (I), respectively. S.E.—Standard error.

	Conversion Model (Linear Regression)									
	Treatments				Model		S.E.	S.E.		
	y-x	n	F	*p*	y = ax + b		a	b	Adj R^2^	β
Prey										
C:N	FR-ET	12	736	<0.017	y = 1.201x	I	0.044		0.894	0.993
	FR-FO	12	104	<0.001	y = 1.154x − 0.854	I	0.113	0.562	0.903	0.955
δ^13^C	FR-ET	12	19,070	<0.001	y = 1.061	S,I	0.008		0.908	0.999
	FR-FO	12	2009	<0.001	y = 1.006x + 1.222	S,I	0.022	0.449	0.995	0.998
δ^15^N	FR-ET	12	2138	<0.001	y = 0.971x	I	0.021		0.903	0.997
	FR-FO	12	2519	<0.001	y = 0.990x	I	0.020		0.904	0.998
Dorsal fins										
C:N	FR-ET	17	7510	<0.001	y = 1.059x	I	0.012		0.935	0.999
	FR-FO	17	23,903	<0.001	y = 0.989x	I	0.006		0.937	0.908
δ^13^C	FR-ET	14	865.0	<0.001	y = 0.918x − 1.617	S,I	0.031	0.466	0.985	0.993
	FR-FO	16	30,169	<0.001	y = 0.969x	I	0.006		0.999	0.999
δ^15^N	FR-ET	15	107,259	<0.001	y = 0.997x	I	0.003		0.928	0.999
	FR-FO	16	50,677	<0.001	y = 1.000x		0.004		0.923	0.999
Newborn										
C:N	FR-ET	20	32,140	<0.001	y = 1.226x	I	0.007		0.999	0.999
	FR-FO	21	22,370	<0.001	y = 0.349x + 2.338	I	0.096	0.384	0.384	0.999
δ^13^C	FR-ET	20	2045	<0.001	y = 0.961x − 2.178	I	0.021	0.407	0.991	0.996
	FR-FO	21	4622	<0.001	y = 1.024x + 1.166	I	0.015	0.317	0.996	0.998
δ^15^N	FR-ET	20	146,531	<0.001	y = 0.984x		0.003		0.999	0.999
	FR-FO	21	12,356	<0.001	y = 0.982x − 0.528	I	0.003	0.315	0.999	0.999
